# Measuring the quality of infection control in Dutch nursing homes using a standardized method; the Infection prevention RIsk Scan (IRIS)

**DOI:** 10.1186/2047-2994-3-26

**Published:** 2014-08-18

**Authors:** Ina Willemsen, Jolande Nelson-Melching, Yvonne Hendriks, Ans Mulders, Sandrien Verhoeff, Marjolein Kluytmans-Vandenbergh, Jan Kluytmans

**Affiliations:** 1 Laboratory for Microbiology and Infection Control, PO Box 90158, 4800 RK, Amphia Hospital, Breda, The Netherlands; 2 Department for Infection Control and Microbiology, St. Elisabeth Hospital, Tilburg, The Netherlands; 3 THEBE, Healthcare Organisation including Nursing Homes, Breda, The Netherlands; 4 Amphia Academy Infectious Disease Foundation, Amphia Hospital, Breda, The Netherlands; 5 Department for Medical Microbiology and Infection control, VUmc Medical Center, Amsterdam, The Netherlands

**Keywords:** Nursing homes, Healthcare associated infections, Antimicrobial resistance, Infection control, Quality improvement, Surveillance

## Abstract

**Background:**

We developed a standardised method to assess the quality of infection control in Dutch Nursing Home (NH), based on a cross-sectional survey that visualises the results. The method was called the Infection control RIsk Infection Scan (IRIS). We tested the applicability of this new tool in a multicentre surveillance executed June and July 2012.

**Methods:**

The IRIS includes two patient outcome-variables, i.e. the prevalence of healthcare associated infections (HAI) and rectal carriage of Extended-Spectrum Beta-Lactamase (ESBL) producing *Enterobacteriaceae* (ESBL-E); two patient-related risk factors, i.e. use of medical devices, and antimicrobial therapy; and three ward-related risk factors, i.e. environmental contamination, availability of local guidelines, and shortcomings in infection prevention preconditions. Results were categorised as low-, intermediate- and high risk, presented in an easy-to-read graphic risk spider-plot. This plot was given as feedback to management and healthcare workers of the NH.

**Results:**

Large differences were found among most the variables in the different NH. Common shortcomings were the availability of infection control guidelines and the level of environmental cleaning. Most striking differences were observed in the prevalence of ESBL carriage, ranged from zero to 20.6% (p < 0.001).

**Conclusions:**

The IRIS provided a rapid and easy to understand assessment of the infection control situation of the participating NH. The results can be used to improve the quality of infection control based on the specific needs of a NH but needs further validation in future studies. Repeated measurement can determine the effectiveness of the interventions. This makes the IRIS a useful tool for quality systems.

## Background

Healthcare associated infections (HAI) constitute a major public health problem [1,2]. Due to the ageing of the population and its associated growing number of people who reside in Nursing Homes (NH), an increase in the burden of HAI is expected [3]. Furthermore, the increase in antimicrobial resistance is a major threat to the safety of elderly people and leads to increased use of broad-spectrum antibiotics [4,5].

To determine the prevalence of- and risk factors for HAI and resistant microorganisms, prevalence surveys are common tools in hospitals and in NH. These surveys typically only include patient-related variables, such as, gender, age, use of medical devices or antimicrobial use [6-8]. However, the concept of infection control is multifaceted and little is known of the importance of ward- or institution specific risk factors for HAI, such as environmental contamination, the availability of local infection prevention guidelines and constraints in infection prevention [9]. These, sometimes overlooked, factors tend to increase the risk of infections among patients during their stay in a NH. Since 2006, a more holistic approach of surveillance has been performed in French hospitals [10]. This surveillances takes a number indicators into account, which are deemed important for reducing infections. The selected indicators include monitoring antibiotic consumption, incidence of surgical site infections, incidence rates of methicillin resistant *Staphylococcus aureus*, yearly consumption of antiseptic hand-wash products and an index of activities for fighting against HAI.

Surveillance is the basis for quality improvement although performing surveillance itself does not result in a change [11]. However, when structured feedback of the surveillance data to Health Care Workers (HCW) and management are given this results in reduced infection rates [12].

Therefore, we developed a tool to provide a standardized and holistic assessment of the quality of infection control in Dutch NH based on a cross-sectional survey. The data were visualised in an easy to read spider plot. We tested the applicability of this new tool in a multicentre study.

## Methods

The IRIS was developed to provide a standardized and holistic measurement of the quality of infection control in health care centres. The data were presented in an easy to read spider plot. The IRIS consists of a cross-sectional surveillance and investigates 2 outcome variables, 2 resident-related risk factors and 3 ward-related risk factors: prevalence of HAI, prevalence of rectal carriage of ESBL producing Enterobacteriacea (ESBL-E), prevalence of medical device, prevalence of antimicrobial therapy, Environmental contamination, shortcomings in infection prevention preconditions and availability of local infection prevention guidelines. The selection of the 5 risk factors was based on the importance, as judged by a group of experienced infection control practitioners, as well as the possibility of an objective and reproducible assessment.

### Setting

The IRIS was performed in 9 NH in the southern part of the Netherlands belonging to one governing organization. Facilities with residents with somatic-, psychosomatic and/or an indication for rehabilitation were included in the survey. The IRIS was performed in June and July 2012.

### Data collection

Two trained infection control practitioners (ICP), collected the data using standardised electronic case record forms. One of the attending NH physicians assisted with the surveillance for HAI, use of medical devices and antimicrobial use. By discussing all (possible) HAI with the NH physician and the other ICP, the results were validated.

The institutional infection control committee and the board of directors of the NH approved the performance of the IRIS. As non-invasive samples were taken and the data was analysed in an anonymous way, informed consent was waived. Participation in the study was voluntary.

### Preparation of feedback data and visualisation

For each outcome variable or risk factor, breakpoints were set to make the division in 3 categories; low, intermediate and high. The breakpoints, for the classification in low, intermediate and high, are based on national prevalence surveys, scientific publications and if no data was available on expert opinion (Additional file 1) [6,7,9].

To visualise all surveillance data in one graphic plot, the data were converted to comparable axis, from 0 up to 100, by an algorithm. The algorithm included the breakpoints for the 3 categories, with low risk from 0 up to 33%; intermediate risk from 34 up to 66% and high risk from 67 up to 100%. Through a pre-programmed excel file, the collected data and the breakpoint for each variable were transformed to a graphic spider-plot on institute level. Each axis of the plot represents an outcome variable or risk factor. If the results were in the high (risk) area, in-depth research and/or improvement activities were recommended, e.g. as antimicrobial use or use of medical devices are situated in the high risk area, the appropriateness of the indication is investigated and reported as well [13].

Population characteristics, which cannot be influenced by HCW activity, are also investigated and used as background information to adjust the interpretation of the risk plot. If the population was classified as high-risk, in-depth research and/or improvement activities were already recommended when results were situated in the intermediate (risk) area. So the interpretation of the spider-plot was partially dependent of the risk profile of the population.

### Population characteristics

All residents present in the NH, on the day of the survey, with a duration of stay in the facility of at least 24 hours, were included. The following population characteristics were assessed: age, gender, admission indication, multimorbidity, pressure ulcer sores and intensity of care needed (scale from zero to 10) [14,15]. Multimorbidity was defined as the presence of two or more chronic diseases [16]. Population characteristics (based on prevalence of multimorbidity, presence of a pressure ulcer sore and mean intensity of care score) were also categorised as low-, medium- and high risk.

### Outcome variables

#### Healthcare associated infections (HAI)

The presence of HAI, sepsis/bacteraemia, lower respiratory tract infections, urinary tract infections, gastrointestinal infections or bacterial conjunctivitis [17], was determined using criteria defined by the Centers for Disease Control and Prevention (CDC) [18]. To be scored as a HAI the resident had to be either symptomatic and/or on antimicrobial treatment, before or on the day of the survey.

The breakpoints, for the classification in low, intermediate and high, of HAI were based on data from the Dutch prevalence surveys for NH [6,7,9].

#### Rectal carriage of ESBL producing enterobacteriaceae (ESBL-E)

Rectal carriage of ESBL-E was determined by culture of perianal swabs (Eswab, Copan Italy) or faeces. Swabs (or faeces) were placed in a tryptic soy broth, containing vancomycin (8 mg/L) and cefotaxime (0.25 mg/L) (TSB-VC). After overnight incubation the TSB-VC was subcultured on an EbSA screening agar plate (AlphaOmega, ’s-Gravenhage, Netherlands), and incubated aerobically overnight [19]. The EbSA agar plate consist of a double MacConkey agar plate containing ceftazidime (1.0 mg/L) on one side and cefotaxime (1.0 mg/L) on the other side.

Species identification and susceptibility testing was performed for all isolates that grew on either side of the agar using MALDI-TOF MS and VITEK 2 (bioMérieux, Marcy l’Etoile, France) respectively. For suspected isolates (MIC ceftazidime and/or MIC cefotaxime > 1 mg/L) the presence of ESBL was phenotypically confirmed with the combination disk diffusion method (Rosco, Taastrup, Denmark), according to the Dutch guideline for the detection of ESBL-E [20]. Genotypic confirmation of the phenotypic ESBL detection was performed using the Check-MDR CT103 microarray (Check-Points, Wageningen, Netherlands) [21].

Reference data on rectal carriage of ESBL-E in NH in the Netherlands are not yet available. This complicates the classification of categories low, intermediate and high). Prevalence of rectal ESBL carriage in 2010/2011 in a large teaching hospital in the same geographical region varied from 4 to 6% with a high variability of the genotypes, indicating that cross transmission is rare with a background prevalence in the population of around 7% [22]. Risk categories were set at below 7% (low risk) and higher than 10% (high risk).

### Risk factors

Resident-, ward- and institution-related risk factors were investigated.

1. Use of medical devices

The presence of medical devices, such as indwelling urethral or suprapubic catheters, intravascular devices, a tracheostomy, a Percutaneous Endoscopic Gastrostomy tube (PEG), gastric tube or Anus Preaternaturalis (AP) stoma, on the day of survey, was registered. The breakpoints, for the classification in low, intermediate and high, of prevalence of medical devices were based on data from the Dutch prevalence surveys for NH [6,7,9].

2. Use of antimicrobial therapy

The use of systemic antimicrobial therapy, on the day of survey, was recorded [13]. Antiviral-, antifungal-, tuberculosis- and inhalation medication, cement beads and topical antibiotic therapy were not included.

The breakpoints, for the classification in low, intermediate and high, of prevalence antimicrobial use were based on data from the Dutch prevalence surveys for NH [6,7].

3. Environmental contamination

Detection of Adenosine Triphosphate (ATP) was used to identify the level of environmental contamination with organic material. The ATP samples were taken using an ATP device (3 M Inc, St. Paul, MN, US) after the routine cleaning in the morning. Samples were taken from 10 pre-defined objects or surfaces within each ward, according to the protocol of the manufacturer (Table 1). The result was expressed in Relative Light Units (RLU). The breakpoints for the classification in low, intermediate and high, of environmental contamination as set by the manufacturer were used (below 1500 RLU clean and above 3000 RLU contaminated) [23,24]. The average ATP value of all results within an institute was presented in the risk plot.

4. Shortcomings in infection prevention preconditions

To initiate a solid infection control policy a number of preconditions are essential. The tested items are listed in Table 1. The average rate of shortcomings within an institute was presented in the risk plot.

5. Availability of local infection prevention guidelines

The Dutch Health Care Inspectorate considers the National infection prevention guidelines as developed by the Dutch Working Group for Infection Prevention as the professional standard. These guidelines have to be adapted and defined to the local setting. We selected 26 infection prevention-related guidelines and checked the local availability in each ward of the institute (Table 1).

The breakpoints, for the classification in low, intermediate and high, for availability of guidelines and shortcomings in precondition were based on expert opinion. The average rate of non-availability within an institute was presented in the risk plot.

**Table 1:** Infection control guidelines checked for availability, tested items environmental contamination and infection prevention preconditions

**Nursing guidelines**	**Guideline concerning bacteria/viruses**	**Tested items environmental contamination**	**Infection prevention preconditions**
Infections in the NH	Multi drug resistant microorganisms	Bathroom sink	Availability of hand alcohol
Patient care	MRSA,	Bedside cabinet	Availability of gloves
Intravenous administration*	Norovirus	Table living room	Availability of FFP2 mouth/nose mask
Medicine administration	Scabies,	Microwave kitchen	Availability of isolation gowns
Cleaning/disinfection and sterilisation	Legionella control,	Medicine cabinet	Availability of needle containers
Storage of sterile materials	Food safety	Bedside commode	Availability of utility room with bedpan washer
Waste collection and transport	Pets in the NH	Utility room	Availability of plastic aprons for employees working in civilian clothes
Urine draining and defecation	Mandatory registration of infectious diseases	Sterile storage shelve	Presence of at least one hand wash basin, per 15 residents
Care of the airways		Toilet seat	Presence of at least two toilet-groups, per 15 residents
Wound care	**Guidelines for employees**	Washing bowl	Presence of at least one single room with private bathroom, per 15 residents
Tube feeding	Hand hygiene		
Dialyses (CAPD/CCPD)*	Personal protective resources,		
Spinal infection procedures and pain management*	Personnel infections		
Care of the airways	Personal hygiene of employees,		
	Blood exposure incidents		

*If applicable.

## Results

**Figure 1:**
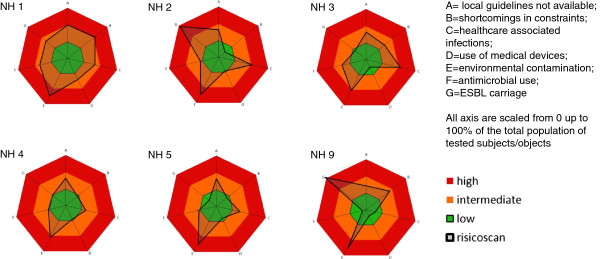
**Infection risk plot per participating NH.** Red = high risk; Orange = intermediate risk; Green = low risk; Black line = IRIS. All axes are scaled from 0 up to 100%. Percentage represent the average rate of non-availability of the 26 guidelines (A), the average rate of non-availability of shortcomings of the 10 preconditions (B), the rate of HAI within the total population (C), the rate of use of medical devices within the total population (D), the average ATP level of all tested subjects/objects (E), the rate of antimicrobial use within the total population (F), the rate of ESBL-E carriage within the screened population (G).

A total of 774 residents in 9 NH were included in the survey (range: 14 – 189 residents per NH). Demographic characteristics are shown in Table 2. The population in the participating NH was in general comparable. Most NH had less than 5% of residents for rehabilitation, while one NH (number 1) had a statistical significant higher proportion (35/174, 20.1%, p < 0.001). The median duration of stay on the day of the survey was 38 days (range 8-428). Significant differences in prevalence of pressure ulcers sore between NH were found; for example 4.4% (8/183) in NH 5 versus 9.8% (17/174) in NH 1 (p = 0.037).Differences were found in outcome variables and risk factors, with a distribution across all 3 risk-categories in the plot. This resulted in different risk-plots for the different NH (shown in Figure 1). The plots from NH 6, 7 and 8 resulted in several axes with no result (0%), due to the low number of residents. These plots are therefore not shown.

**Table 2:** Characteristics of the resident population

**Nursing home**	**N**	**Mean age (Median)**	**Nursing home indication**			**Mean intensity of care (score 1-10)**	**Prevalence of multimorbidity (%)**	**Prevalence of pressure ulcers (%)**
			**Psycho-geriatric**^**#**^	**Rehabilitation**^**#**^	**Somatic**^**#**^			
1	174	74.3 (78)	93 (53.4%)	35 (20.1%)	46 (26.4%)	6.295	172 (98.9%)	17 (9.8%)
2	35	84.7 (85)	26 (74.3%)	0	9 (25.7%)	6.114	35 (100%)	0
3	63	84.1 (85)	51 (81.0%)	3 (4.8%)	9 (14.3%)	5.270	56 (88.9%)	0
4	74	83.9 (84)	60 (81.1%)	0	14 (18.9%)	5.703	72 (97.3%)	7 (9.5%)
5	183	71.5 (78)	101 (55.2%)	7 (3.8%)	75 (41.0%)	6.005	176 (96.2%)	8 (4.4%)
6	17	88.4 (90)	14 (100%)	0	3 (17.6%)	5.059	17 (100%)	0
7	14	82 (82)	14 (100%)	0	0	5.714	14 (100%)	2 (14.3%)
8	25	80.6 (82)	25 (100%)	0	0	4.960	24 (96.0%)	1 (4%)
9	189	79 (81)	185 (97.9%)	0	4 (2.1%)	5.644	167 (88.4%)	14 (7.4%)
Total	774	77.6 (82)	569 (73.5%)	45 (5.8%)	160 (20.7%)	5.838	733 (94.7%)	49 (6.3%)

^#^The NHs were divided in separate ward for psychogeriatric, somatic and rehabilitation indication. A patient with somatic and rehabilitation indication was categorised depending on the kind of ward.

A total of 59 (7.6%) residents had at least one medical device present on the day of the survey (ranging from 0% up to 12.1% in the different NH). The highest prevalence of medical devices was observed in the NH with a high proportion (20%) of rehabilitation residents. The most prevalent medical device was a urethral catheter (n = 42), next a suprapubic catheter (n = 9), a PEG (n = 7), a tracheostoma (n = 4), one peripheral intravascular catheter and two others.

**Figure 2:**
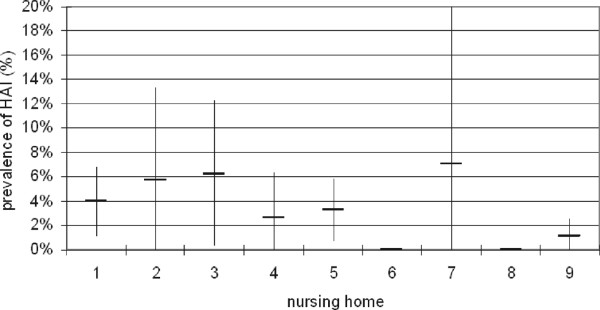
Prevalence of healthcare associated infections in the 9 participating NH (vertical bars represent the 95% confidence interval).

**Figure 3:**
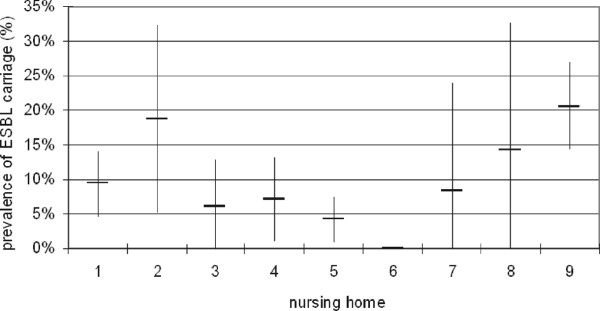
Prevalence of rectal carriage of ESBL in the 9 participating NH (vertical bars represent the 95% confidence interval).

Forty (5.2%) residents were treated with antimicrobials on the day of the prevalence survey. Nitrofurantoin and amoxicillin/clavulanic acid were most frequently used (8 and 7 times, respectively). Eighteen residents (45% of all residents on antimicrobial therapy) were treated with antibiotics while no HAI was registered. The indications for antimicrobial use were skin infection (n = 5), respiratory infections (n = 2), surgical site infection already present on admission (n = 2), prophylaxis (n = 9), which include maintenance dose for recurrent urinary tract infections (n = 6), when changing a catheter (n = 1), maintenance dose for COPD (n = 1) and unknown (n = 1). Differences of antimicrobial use in the NH were observed (range 0% - 9.2%). NH 9 had a significantly lower prevalence of antimicrobial use than the average use in the other NH (*p* = 0.002).There was no significant difference in the prevalence of HAI between the NH (Figure 2), with an overall mean prevalence rate of 3.1% (n = 24). Urinary tract infections (n = 18) and lower respiratory tract infections (n = 5) were the most frequently observed HAI.A total of 643 (83.1%) of the 774 residents were tested for rectal ESBL carriage. The main reason for non-responding was lack of interest. Of those who were screened, ESBL was detected in 70 (10.9%), ranging from zero to 20.6% in the different NH. The highest rates were found in NH 2 and 9 (Figure 3, p < 0.001). In all NH with an ESBL carriage rate (classified as intermediate or high - orange or red area), spread of a specific ESBL clone was identified.

On average, 24% of the investigated infection control guidelines were not available (range 19% - 50%). Infection control preconditions were missing in all NH: overall 18% with a range from 5% in NH 2 to 40% in NH 8. In all but 2 wards, hand alcohol was available. Most frequently observed shortcomings were the absence of protective gowns and the absence of at least one hand washing facility per 15 residents.

Environmental contamination was high in all but one NH (NH7). Heavily contaminated surfaces were most frequently found on the bedside commode (58/59 = 98.3%), kitchen/microwave (34/56 = 60.7%) and toilet seat (36/62 = 58.0%).

The surveillance could be completed in about 2 hours per department (approximately 20 residents). Validation, analyses, preparation of feedback data and visualisation took another 2 hours. The total time investment for performing an IRIS in these 9 NH (774 residents) was 20 days.

All surveillance data and plots were presented to-, and discussed with management of the governing organisation. Subsequently every IRIS plot was presented to management and healthcare workers of the involved institute. The message was well received by management and an extensive improvement program was initiated; infection preconditions were resolved, missing guidelines were developed and implemented and an education program has started. The NH with the high prevalence of ESBL-E carriage informed the inspectorate for healthcare about the findings and initiated and outbreak management team to contain the spread of the outbreak strain.

## Discussion

The IRIS showed a large variation in outcome variables and risk factors among the 9 participating NH. The management of the NH used the information to initiate targeted improvements of the infection control situation. The common shortcomings were the availability of infection control guidelines and the level of environmental cleaning. The most striking differences were found in the prevalence of ESBL. The extremely high ESBL carriage rates in some NHs deserve further investigations. In NH 9, extensive clonal spread of *E. coli* ST131, a well-known epidemic clone associated with health care facilities, was found [25]. These results will be published separately. Before the IRIS was performed none of the NH was aware of a problem with ESBL. In general, NH do not perform microbiological investigations frequently and most antibiotic prescriptions are given empirically without knowledge of the pathogens involved and their antibiotic susceptibility. It is likely that extensive antibiotic exposure, close contact with other antimicrobial-exposed individuals, age and health-associated alterations in intestinal microbiota all contribute to the high prevalence of multidrug resistant bacteria among the elderly population [22]. We deliberately included an active surveillance for resistant pathogens in the IRIS to qualify and quantify this issue. The geographic area of the NH in our survey is close to the Belgian border, where prevalence rates from 0 up to 20% have been described by Jans et al. [26]. There is no physical border between the two countries and a lot of people in this area travel across the border on a daily basis. On the other hand the healthcare systems are clearly divided and almost all Dutch residents will be cared for in a Dutch Nursing home and the same goes for Belgium. The NH with high rates in our survey were on the upper limit of what was found in Belgium. IRIS showed that resistance may be a relevant issue in The Netherlands as well and active monitoring should be seriously considered. ESBL-E carriage proved to be an important indicator to include in a risk assessment for infection control.

There were no significant differences between prevalence of HAI and antibiotic use between the individual NH, except for one outliner regarding antimicrobial use. More research is needed to investigate the appropriateness of therapy. However, the data already suggest frequent inappropriate use of antimicrobial therapy, in particular the use of prolonged maintenance doses.

The reliability of data collection and interpretation, especially when more than one person is involved, is critical for the reproducibility of IRIS. To obtain reliable data we recommend to validated all (possible) HAI with the NH physician and at least one ICP or other dedicated and trained professional.

The IRIS has several limitations. First, skin and/or soft tissue infections (SSTI) were not recorded in this survey, as these were not included in the current method of the national surveillance initiative [17]. However, during the survey the ICP, repeatedly noted the presence of a SSTI. The described prevalence can therefore be an underestimation of the real HAI prevalence. Based on our observations we will include these infections in future IRIS. The use of the McGeer definitions for HAI infections was considered, however there were two reasons to ultimately decide against them [27]. Namely, the McGeer definitions required detailed information that was not registered and available on a daily base in the NHs of our survey. Furthermore, the definitions are not frequently used in the Netherlands and therefore it was not possible to achieve local reference data to set breakpoints for the classification in low, intermediate and high, or to compare our results with other Dutch prevalence rates.

Second, the breakpoints for three categories were based on limited experience and expert opinion. This is largely an arbitrary decision that should be evaluated and adjusted when necessary in the future. Third, not all residents were screened for ESBL-E carriage, which is a potential cause for bias. Overall 83% was screened including all departments of the NHs so we consider this of limited importance. Fourth, the variables included in the IRIS are limited. Hand hygiene compliance was not measured in this survey due to the time investment it takes to perform adequate compliance observations. In retrospect this is a missed opportunity that will be added to the IRIS in the future.

Furthermore, it can be considered to include the turnover of HCW, the staffing level and the education level of the staff in the model. Others have found that these factors are associated with the occurrence of HAI in NH [28].

Finally, results from the smaller NH should be interpreted carefully due to the limited sample size. In this case the high prevalence rates have to be confirmed by additional measurements. We therefore recommend performing the IRIS only in NH with more than 50 residents and even in those settings a single measurement should be interpreted with caution. Also, the breakpoints for some measures, e.g. HAI, are relatively narrow considering the random-variation of the point prevalence estimates.

## Conclusion

The IRIS provided a relatively rapid and complete view of the current state of infection control in NH. There was substantial variation between the different NH and this provided different conclusions for the individual NH. The visualisation in a risk plot was helpful to provide feedback because it was easy-to-read and explained itself to a large extend. Feedback of the IRIS was the trigger to start an extensive improvement program on multiple fields. Whether the results obtained by the IRIS will eventually lead to measurable reduction in HAI requires further studies.

The most remarkable finding were the striking differences in ESBL carriage rates between the NH. These outbreaks are currently under investigation for the sources and transmission routes. The effects of the improvement program can be measured in a repeated measurement. In that way a quality control circle with continuous improvement can be achieved. Therefore, the IRIS can be a valuable tool to provide a holistic assessment of infection control and quality improvement focusing on infection control.

## Abbreviations

AP: Anus preaternaturalis; ATP: Adenosine triphosphate; CDC: Center for disease control and prevention; ESBL: Extended spectrum beta-lactamase; ESBL-E: Extended spectrum beta-lactamase producing enterobacteriaceae; HAI: Healthcare associated infections; HCW: Healthcare worker; ICP: Infection control practitioner; IRIS: Infection control risk scan; NH: Nursing home; PEG: Percutaneous endoscopic gastrostomy; SSTI: Skin soft tissue infection; TSB-VC: Tryptic soy broth containing vancomycin and cefotaxim.

## Competing interests

All authors report no conflicts of interest relevant to this article.

## Authors’ contributions

IW, JN, YH and JK designed the study. JN and YH assembled all input data. AM and SV coordinated and supported the communication within the study setting. IW, MK and JK analysed the data. All authors discussed the results and implications. IW and JK wrote the manuscript. JN, YH, AM, SV and MK commented on the manuscript at all stages. All authors read and approved the final manuscript.

## Supplementary Material

Additional file 1 Characterisation of the resident population and risk classification of the infection risks: low-, medium- and high risk. Click here for file
