# Supplementary material for: Measuring the quality of infection control in Dutch nursing homes using a standardized method; the Infection prevention RIsk Scan (IRIS)
Source: Antimicrob Resist Infect Control. 2014 Aug 18;3:26. doi: 10.1186/2047-2994-3-26 (PMC4169692; doi:10.1186/2047-2994-3-26)
Supplement: Additional file 1 — Characterisation of the resident population and risk classification of the infection risks: low-, medium- and high risk. [file 2047-2994-3-26-S1.doc]

**Table 1** Characterisation of the resident population and risk classification of the infection risks: low-, medium- and high risk

|  | Low | Intermediate | High | Reference data |
| --- | --- | --- | --- | --- |
| Resident population |  |  |  |  |
| prevalence multimorbidity | <15% | ≥15 and <30% | ≥30% |  |
| prevalence pressure ulcer(s) | <10% | ≥10 and <25% | ≥25% |  |
| intensity of care (scale) | 1-4 | ≥ 5 and < 7 | ≥ 8 |  |
|  | Low | Intermediate | High |  |
| Outcome variables |  |  |  |  |
| prevalence of HAI& | <2% | ≥ 2 and < 5% | ≥5% | PREZIES: 4.2% |
|  |  |  |  | Eilers et al: 2.8%, range between the NH: 0.10-5.6% |
|  |  |  |  | Eikelenboom et al: 6.7%, 95%CI 5.4-8.2% |
| rectal carriage of ESBL-E^#^ | <7% | ≥ 7 and <10% | ≥10% | Nearby Dutch hospital: 4 to 6% |
|  |  |  |  | Jans et al: 12.2 (ranged 0-36%) [Belgium] |
|  | Low | Intermediate | High |  |
| Risk factors |  |  |  |  |
| the use of medical devices | <10% | ≥10 and <15% | ≥15% | PREZIES: urethrakatheter 7.5%, 95% CI 3.9-8.2 |
|  |  |  |  | Eilers et al: urethrakatheter 12% |
|  |  |  |  | Eikelenboom et al: urethrakatheter 5.1%, range 3.8-6.8 |
| prevalence of antimicrobial therapy | <5% | ≥5 and <10% | ≥10% | PREZIES: 6% |
|  |  |  |  | Eikelenboom et al: 6.6%, range between the NH 5.5-7.3 |
| environmental contamination | < 1500 RLU* | ≥1500 and < 3000 RLU | ≥ 3000 RLU | Manufacterer: grey zone 1500-3000 RLU |
| availability of local guidelines | <10% not-available | ≥10 and <70% not-available | ≥ 90% non-available | Expert opinion |
| shortcomings in infection prevention related preconditions | <10% shortcomings | ≥10 and <70% shortcomings | ≥ 70%shortcomings | Expert opinion |

^#^ESBL-E = Extended Spectrum β-lactamase producing Enterobacteriaceae.

&HAI = Healthcare Associated Infections; ESBL = Extended Spectrum Beta-Lactamase.

*RLU = Relative Light Units.
